# 

**Published:** 1905-01

**Authors:** 


SUBSCRIPTION $1.00

PUBLISHED MONTHLY

### ATLANTA JOURNAL-RECORD OF MEDICINE.

Successor to Atlanta Medical and Surgical Journal, Established 1855, and Southern Medical Record, Established 1870.

VOL. VI. ATLANTA, GA., JANUARY, 1905. No. 10.

